# Biobanking human embryonic stem cell lines: policy, ethics and efficiency

**DOI:** 10.1007/s40592-015-0050-y

**Published:** 2016-01-13

**Authors:** Søren Holm

**Affiliations:** Centre for Social Ethics and Policy, School of Law, University of Manchester, Manchester, M13 9PL UK; Centre for Medical Ethics, University of Oslo, Oslo, Norway; Department of Health Science and Technology, Aalborg University, Aalborg, Denmark

**Keywords:** Commercial interests, Embryo, Embryo donation, Exploitation, Gamete donation, Human embryonic stem cells, Stem cell banks

## Abstract

Stem cell banks curating and distributing human embryonic stem cells have been established in a number of countries and by a number of private institutions. This paper identifies and critically discusses a number of arguments that are used to justify the importance of such banks in policy discussions relating to their establishment or maintenance. It is argued (1) that ‘ethical arguments’ are often more important in the establishment phase and ‘efficiency arguments’ more important in the maintenance phase, and (2) that arguments relating to the interests of embryo and gamete donors are curiously absent from the particular stem cell banking policy discourse. This to some extent artificially isolates this discourse from the broader discussions about the flows of reproductive materials and tissues in modern society, and such isolation may lead to the interests of important actors being ignored in the policy making process.

## Introduction

This paper will identify, analyse and discuss a number of arguments each providing a possible full or partial justification for the establishment and maintenance of human embryonic stem cell banks^1^ from which researchers can easily obtain a variety of stem cell lines for research purposes. It will look at the relative importance of these arguments in policy discussions leading to the establishment of stem cell banks, and in later policy discourses related to the continued public and professional legitimacy of the banks. The arguments to be analysed are of two types. First, ‘ethical arguments’ that pertain to the protection of embryos, and the protection of embryo and/or gamete donors. Second, ‘efficiency’ arguments include any and all of the following: an interest in easy access to human embryonic stem cells (hESC) for research; efficient promotion of stem cell research, encompassing standardisation and quality control (including the standardisation of ‘ethics’); and standardised conditions of transaction, simple application procedures, reasonable prices, and non-onerous intellectual property (IP) terms. Although there is some overlap, especially in the area of arguments relating to the benefits of standardisation of ethical requirements for embryo and gamete donation for stem cell derivation, these categories are nevertheless distinct. Many of the particular examples are drawn from debates in the UK, but where relevant and necessary material from other jurisdictions is also discussed.

In debates about stem cell banks these arguments are often used jointly as when Baroness O’Neill (the philosopher Onora O’Neill) stated in the UK House of Lords that “the creation of the Medical Research Council (MRC) stem cell bank will be a good way to support regulated and effective research and to minimise the use of early embryos” (Hansard 2002a). Lord Hunt, the responsible government minister added that “The noble Baroness, Lady O’Neill, referred to the establishment of a national stem cell bank, the first of its kind. This is very important. It will curate ethically-sourced quality controlled stem cell lines for research leading to the development of new therapies. It will provide access to standardised reagents, reduce the need for individual groups to generate their own cell lines and will reduce the numbers of embryos used for embryonic stem cell research” (Hansard 2002b).In both interventions arguments related to minimisation of the use of embryos are mentioned alongside arguments about the importance of a stem cell bank in promoting research. A similar conjunction of reasons for stem cell banking can be found in the current UK Stem Cell Bank “Code of practice for the use of human stem cell lines”:

The UK Stem Cell Bank facilitates the sharing of quality controlled stem cell lines by the clinical and research communities. In so doing [sic] supports research that will help to better understand human development and disease and aid the development of strategies to treat serious diseases. It will also reduce the need for individual research teams to generate their own stem cell lines, minimise the use of human tissues, and enable different researchers to work on identical material so that direct comparisons may be made between studies. (UK Stem Cell Bank Steering Committee 2010: 14).

The legal establishment of the Spanish Banco Nacional de Lineas Celulares also mentions a range of principles underlying the formation of the bank:

Artículo 3. Principios.

El Banco Nacional de Líneas Celulares actuará de acuerdo a los principios éticos recogidos en los convenios internacionales en materia de biomedicina y derechos humanos de los que España sea parte y de acuerdo con los principios de objetividad, colaboración, integración y solidaridad (Orden SCO 2006).^2^

The primary interest in this paper is in the arguments made and how they are employed in debates about public policy in policy making fora, whether these fora are overtly political or more technocratic. The prevalence and evaluation of these arguments in the academic literature is only of secondary relevance. The arguments have been identified from an analysis of policy debates relating to the establishment and maintenance of the UK Stem Cell Bank and the US National Stem Cell Bank and from publications and other materials from the International Stem Cell Banking Initiative and the International Society for Stem Cell Research. Each of these arguments directs the attention of the policymaker to a particular set of concerns, interests and actors as objects of protection and/or promotion, and may if considered or used in isolation obscure other concerns, interests and actors. It is therefore of interest to identify both the arguments that are made and those that are not made in the public debates. It is also of interest to interrogate whether the arguments are cogent in the form in which they are put forward. Although the discussion will primarily focus on stem cell banking for research, issues related to commercialisation and therapy will be briefly discussed in the penultimate section of the paper.

## Background

A number of non-commercial and commercial stem cell banks have been established from which researchers can source stem cells for research. The services provided by these banks, and the conditions under which researchers can obtain the cells vary widely, but all of the banks act as intermediaries between the researcher and the current holder(s) of the IP and non-IP rights in the stem cell lines held by the bank (Isasi et al. 2009; Hug 2009). Some stem cell banks only hold cell lines derived at one specific institution, whereas others hold lines from many institutions in a variety of jurisdictions. The two largest stem cell banks, WiCell in the USA and the UK Stem Cell Bank, both hold cell lines from many different institutions. Stem cell banks differ from stem cell registries in that the banks physically hold and distribute the stem cell lines, whereas stem cell registries merely hold information about the cell lines.

A decision to deposit a stem cell line in a stem cell bank can be either voluntary or mandatory. In the UK researchers who derive a stem cell line from a human embryo are required to deposit the line in the UK Stem Cell Bank, because the Human Fertilisation and Embryology Authority inserts this as a requirement in any license to derive stem cells. But the UK Stem Cell Bank also curates cell lines from outside the UK, as well as Induced Pluripotent Stem cell lines and standardised feeder cell lines. And UK derived cell lines can be deposited in banks outside of the UK, for instance in WiCell. A similar mandatory deposit scheme operates for the Spanish Banco Nacional de Líneas Celulares (International Stem Cell Forum). Other countries, such as Canada, have established mandatory registration of stem cell lines derived with public funding, but do not operate a bank as such (CIHR 2014).However, most jurisdictions do not have mandatory deposit or registration schemes and banks in these jurisdictions are private institutions. This includes major stem cell deriving jurisdictions such as the USA, Australia, India and China.

The US had a National Stem Cell Bank sponsored by the National Institutes of Health (NIH) from 2005 to 2010. It was established as part of WiCell and its explicit purpose was to ensure access for US researchers to the 21 cell lines eligible for NIH funding following President Bush’s Presidential Statement on stem cell research funding on August 9, 2001 (Bush 2001).

## Protection of the embryo

As already alluded to above in the quotes from the 2002 UK House of Lords debate, one possible justification for establishing a stem cell bank is that it will reduce the number of embryos that are destroyed in order to derive stem cell lines, and that this is important because it shows respect for the value (or worth) of human embryos. This argument was particularly important in the UK debate where it has been widely accepted in the political discourse that although embryos are not full human beings with rights, they have a special status, should command respect and should only be created or used for important purposes. This argument goes back to the Warnock Committee report published in 1984:

Nevertheless we were agreed that the embryo of the human species ought to have a special status and that no one should undertake research on human embryos the purposes of which could be achieved by the use of other animals or in some other way. The status of the embryo is a matter of fundamental principle which should be enshrined in legislation. *We recommend that the embryo of thehuman species should be afforded some protection inlaw* (Committee of inquiry into human fertilisation and embryology 1984: 63, emphasis in original).

And a version of the respect argument has more recently been defended by Sandel:

Those who view embryos as persons often assume that the only alternative is to treat them with moral indifference. But one need not regard the embryo as a full human being in order to accord it a certain respect. To regard an embryo as a mere thing, open to any use we desire or devise, does, it seems to me, miss its significance as potential human life. Few would favor the wanton destruction of embryos or the use of embryos for the purpose of developing a new line of cosmetics. Personhood is not the only warrant for respect. For example, we consider it an act of disrespect when a hiker carves his initials in an ancient sequoia—not because we regard the sequoia as a person, but because we regard it as a natural wonder worthy of appreciation and awe. To respect the old-growth forest does not mean that no tree may ever be felled or harvested for human purposes. Respecting the forest may be consistent with using it. But the purposes should be weighty and appropriate to the wondrous nature of the thing (2004, p. 208).

Sandel’s version of the respect argument targets what could be called the dichotomy view of the moral status of the embryo, i.e. either it has full moral status or it has no moral status. He ascribes this view to those who view embryos as persons, but it is as commonly held and argued for by those who view embryos as non-persons. Like many intermediate positions (think, for instance of the common gradualist arguments in relation to the moral status of the fetus) the respect argument will be criticised by both sides and is unlikely to convince either. As a strictly philosophical argument the respect argument is therefore problematic in the sense that whereas it accords well with fairly commonly held intuitions in societies with a Christian cultural history, it is difficult to provide it with a convincing philosophical underpinning. However, it has an important place in public debate because it resonates with common intuitions and because it allows policy makers to pursue liberal policies while (seemingly?) espousing conservative values. In many jurisdictions policy makers cannot state that they think that human embryos have no intrinsic value and can be used for research or stem cell derivation without any safeguards. But, by couching their considerations in the language of respect they can pursue and promote stem cell derivation and banking while still claiming to respect the value of the embryo.

Along these lines, easily accessible stem cell banks are thought to reduce the number of embryos created and used for stem cell derivation because they allow researchers who do not have a need for a very specific type of stem cell to obtain stem cells without having to derive them directly from embryos. Stem cell banks mediate between stem cell derivers and stem cell users and help to maximise the use of those stem cell lines that are held in the bank. Stem cell banks holding cell lines derived to good manufacturing practice (GMP) standards may, in the future, hold the same mediating role between stem cell derivers and commercial entities that require stem cells as the basis for the production of a cell-based product or therapy.

Whether the establishment of stem cell banks have actually reduced the number of embryos used for stem cell derivation is an empirical question, but an empirical question it is almost impossible to answer conclusively either way. For reasons unrelated to stem cell banking, the research field has focused on and used a limited number of stem cell lines (Scott et al. 2009). And, the invention of induced pluripotent stem cells in 2006/2007 has meant that the research and development trajectory of the field has changed fundamentally from what was envisaged when stem cell banks were first set up.

## Protection of embryo and/or gamete donors

A separate argument for the establishment of stem cell banks is that they have a role in protecting embryo and/or gamete donors. They arguably do this in two distinct ways (1) by reducing demand for embryos and gametes, and (2) by fostering ethical practice in donation or acquisition. Two things are important to note here. First, I use the terminology’ donation or acquisition’ because it makes little sense to claim that embryos or gametes that are acquired for valuable consideration are donated (i.e. given as a free gift or as part of a gift exchange). On the other hand, embryos and gametes are often acquired in ways where money does not change hands, but where it makes sense to nevertheless see the transaction as akin to buying and selling. Second, the limb of this particular argument referring to a reduced demand for embryos and gametes does not rely on any view of the moral status of the embryo, since the concern here is not with the embryo or the gametes, but with the effect of donation/acquisition practices on women and men (i.e. full human persons on anybody’s account) participating in these practices.

As discussed above stem cell banks may help to reduce the number of embryos that are used for stem cell derivation, and this will in turn reduce the need for donation/acquisition. Fewer couples or single women will be asked to donate embryos for stem cell derivation, and fewer women asked to donate or sell their ova for the creation of embryos.^3^ This can be seen as important because (1) donation decisions are often complex and difficult (Haimes and Taylor 2009), and (2) female gamete donors and sellers have to undergo non-trivial medical procedures and may in some circumstances also be the victims of exploitation. Buying and selling ova does not *necessarily* involve exploitation and not all exploitation is illegitimate. We can for instance imagine a situation where a rich and powerful woman sells her ova to stem cell derivers because she believes in the good of stem cell research and where she would have donated without payment if asked. But, even though the practice of buying and selling ova does not necessarily involve exploitation, it does often exploit the poor and vulnerable in ways that are, at least *prima facie* illegitimate (Baylis and McLeod 2007; Rao 2006; Widdows 2009).

Couples and single women are asked to donate embryos for research in a range of different contexts, with varying complexity and ethical problematics. At one end of the spectrum is the couple or single woman being asked to donate an embryo that is truly and finally ‘spare’, i.e. an embryo that is still in storage after the couple or single woman have completed their reproductive project.^4^ At the other end are decisions where the reproductive project is still ongoing, or perhaps only starting as in the case of embryo-sharing arrangements wholly or partially funding IVF procedures.^5^ There is good evidence that contemplating a donation decision is not only complex but also stressful for some potential donors. This entails that whatever view we take on the ethics of the different forms of embryo donation, we have an interest in only asking people to donate if their donation is necessary. And this derivatively creates an interest in reducing the number of donation requests to the level that is necessary for the conduct of stem cell science.

There is large literature on the ethics of donation/acquisition of ova, and it is not necessary to rehearse it in detail here (Baylis and McLeod 2007; Waldby 2008; Widdows 2009; Haimes et al. 2012; see also Haimes and Taylor, this issue, and Baylis and Widdows, this issue). Suffice it to say that ova for research purposes are generally not acquired through donation, but through some form of acquisition for valuable consideration. They may either be bought outright for money, or they may be bought indirectly through egg sharing arrangements where ova are given up in exchange for IVF treatment. Both the direct and the indirect buying of ova raise potential issues of exploitation, and this provides reasons to regulate and minimise these practice sunless ways can be found to make them’ exploitation proof’.

Stem cell banks may also contribute to the protection of embryo and gamete donors by setting and enforcing ethical standards in relation to donation/acquisition of embryos and gametes for stem cell research. If stem cell banks define ‘ethical’ standards for donation/acquisition, and make these standards a requirement that must be met as part of the process for accepting a stem cell line into the bank, then this may foster good ethical practice (insofar as the standards are good ethical standards).^6^ Derivers of stem cells have an interest in having their stem cell lines widely available through stem cell banks and thereby an interest in meeting the requirements of the banks. This may be a strong interest if the presence of a stem cell line in a certain stem cell bank or set of banks come to be seen as a signal of quality, or if it leads to much wider use of the line.^7^ In jurisdictions where donation/acquisition is regulated and controlled the standard setting influence of biobanks may be limited. However, stem cell derivation also takes place in jurisdictions without significant regulation or where regulations are not enforced, and in such jurisdictions the standard setting by biobanks may be more important.

This ethical standard setting function can be further enhanced by international ‘best practice’ guidelines for stem cell banking that incorporate ethical considerations as part of ‘best practice’ (International Stem Cell Banking Initiative 2009; Crook et al. 2010).However, a closer analysis at the ethical best practice guidelines that are proposed show that they are primarily concerned with ensuring that the consent to embryo donation is fully informed and voluntary, and ignore many other ethical issues such as the potential for exploitation.

## Efficiency, quality and the promotion of stem cell research

Let us accept, for the sake of argument, that stem cell research is important both scientifically and because it will lead to important new therapies. And let us further accept that some of these benefits are necessarily linked to hESC research, i.e. they cannot be achieved through other forms of research.^8^ If we accept these premises it becomes important that structures are put in place that facilitate the conduct of high quality stem cell research, and stem cell banks are arguably one of these structures.^9^

Stem cell banking can facilitate high quality research in a number of different ways. The technical work done by stem cell banks when cell lines are accepted for curation and later distributed is important in this respect. A researcher who obtains a cell line from a well-run stem cell bank can be reasonably sure that it is the correct cell line, that its provenance is known and that if s/he follows the instructions the cell line will grow. Stem cell banks thus help the stem cell field to avoid some of the problems that have plagued cell culture in the past.

It is well documented in the literature that there have historically been problems with contamination of human and animal cell lines commonly used for research (Lacroix 2008; Capes-Davis et al. 2010). A significant number of lines have, for instance, been contaminated with the HeLa cell line. This means that research results may be inaccurate or false because researchers have really been working with HeLa cells and not with the cells they thought they were working with (Stacey 2000). But the quality control measures of a well-run stem cell bank ensure that the cell line that is distributed is the right one. Stem cell banks also do considerable work in understanding the growth requirements of each specific cell line, in order to ensure both that the bank can propagate the line and that downstream users can successfully grow it in their own labs. This function may be especially important for users who use stem cells primarily as a research tool and who are not expert in (human) cell culture.

Another important facilitative feature of stem cell banks is that they make access easier for stem cell users. Instead of having to locate the multiple holders of the IP and other rights in relation to stem cell lines,^10^ the users have a one-stop shop. The UK Stem Cell Bank, for instance, currently provides access to 25 hESC lines, on behalf of 10 different right holders, from 4 different continents.^11^ And all 25 lines are provided under a uniform Research Use License and Material Deposit and Distribution Agreement.^12^ This means that a user only has to deal with one, uniform set of legal documents in order to get access to a large number of cell lines. WiCell operates with a different model in relation to IP rights. It allows the owners of the rights to set their own terms, but these terms are readily accessible through WiCell’s stem cell ordering system and the end-user does not have to negotiate with the owners of the rights. Stem cell banking thus creates a very significant reduction in the transaction costs of stem cell research.

## Arguments and legitimacy

Which of the arguments discussed above are most important for the legitimacy of, and continued public/political/scientific community support for stem cell banking? In the UK context, it seems the two most important arguments justifying the establishment of the UK Stem Cell Bank were the protection/special status of the embryo, and the efficiency arguments. In-depth ethnographic research by Neil Stephens has shown that the UK Stem Cell Bank is placed in a very complex landscape of stakeholders which it has to navigate in order to secure continued support (Stephens et al. 2008a, b, 2011). In this landscape the efficiency arguments are very important, but equally important is a factor that has not been considered here so far: that the existence of the bank, with an independent governance structure, provides public legitimacy to the UK stem cell endeavour by allowing researchers and policymakers to claim that UK stem cell derivation and research is governed within a robust ethical framework. In the US, the efficiency arguments played a significant role in the National Institutes of Health decision to fund the establishment of the National Stem Cell Bank, but it is probably not these arguments that are most important for the continued support for the bank. In particular, the change from the Bush to the Obama administration and the concomitant shift in stem cell policy meant that the efficiency arguments supporting the National Stem Cell Bank became less important and the NIH reverted to a stem cell registration system.

The precise conjunction of arguments that have supported the establishment of other stem cell banks varies, but despite extensive literature searches including searches of relevant news media and official documents I have not been able to find any evidence that considerations relating to donors or sellers of embryos or gametes have played a major role. This is surprising, since embryo and, especially female gamete donors and sellers are those whose welfare and rights are most directly affected by stem cell derivation and banking.^13^ The donors and sellers are involved bodily, and they are the persons who are asked to waive rights in the derivation and banking process. Not only do they give up their property and other rights in the actual reproductive materials that they donate and sell, they also in practice give up the right not to have (50 % of^14^) their genome genotyped or sequenced without their knowledge and consent. Characterisation of stem cell lines already involves genotyping, and is in the future likely to involve genome sequencing as well. It could be counter argued that all of our welfares are bound up with stem cell research, if stem cell research can successfully deliver on its promises, but then we are not affected now, only possibly in the future.

## Future challenges

Most stem cell banks have until now focused on curating and distributing stem cell lines for research, but as the commercialisation of stem cell therapies come closer new challenges emerge that threaten the legitimacy of the stem cell banking enterprise. The first of these challenges arise because the owners of the rights relating to stem cell lines may wish to restrict access to these lines, and not allow wide distribution for research purposes. There may be an interest in restricting or completely barring access if a cell line is used as the basis for a specific product. In that context an ‘adverse finding’ in relation to that particular stem cell line may lead to delays in the regulatory process and further lead to later introduction to the market and significant economic loss. Barring access to the line may also make it less likely that competitors can develop a biosimilar product. But restricting access of *bona fide* researchers to a stem cell line goes against all of the most important justifications for establishing stem cell banks for research, unless it can be plausibly argued that there are already ‘as many and as good’ lines available so that not being able to use this particular line does not affect the progress of stem cell science in any way. But such an argument is *prima facie* implausible, since there is likely to be something special about the cell line for which the owners want restricted access. It is likely to have been chosen as the basis for product development exactly because it is not average. It may, for instance, be easier to differentiate to a particular cell type than the average line.

In jurisdictions where stem cell banks are private entities this mismatch between a depositor demand for exclusivity and the standard justifications for stem cell banking does not create a significant problem for the stem cell banks. They can just refuse to curate such lines. However, in countries with mandatory deposit requirements, demands for exclusivity create a more acute problem. Not only is there the mismatch identified just above, but curating a line in the bank that is restricted to only one user is, furthermore, financially irrational, unless the user pays directly for the curation. Otherwise the bank is simply supporting a particular commercial entity by curating the cell line on its behalf, without there being any public gain in terms of increased research possibilities. But if there is a legal mandatory deposit requirement the bank is placed in a difficult position if it refuses to accept a line, and a standoff may develop between the bank and the depositor. If demands for exclusivity became common a system of stem cell banking with mandatory deposit requirements may come under severe strain, because granting exclusivity undermines both the ethical and the efficiency arguments supporting the system.

Another and more fundamental challenge to the current stem cell banking model arises if and when stem cell science develops to a stage where the ‘individualised stem cell therapy’ promissory becomes realistically implementable. At that point we are going to need a completely different type of stem cell bank, but a discussion of the new ethical issues that this will raise is beyond the scope of this paper (Faden et al. 2003).

## Conclusion

This paper has shown that the establishment and continued public and professional legitimacy of human embryonic stem cell banks rests on a multiplicity of supporting justifications. Some of these are more important and cogent in relation to the establishment of such banks, and some in relation to the ongoing functions of the banks. The specific conjunction of justifications relevant to different banks may vary according to local context, and the conjunction may not be stable over time. Reasons and justifications that seem important at one time, may later come into conflict with new functions of a bank. In general ‘ethical reasons’ are important for the establishment of banks, whereas ‘efficiency reasons’ are more important for the continued existence of the banks.

Perhaps more importantly the paper has also identified an interesting lacuna within what can broadly be called the ‘ethical justifications’ for stem cell banking. Stem cell banks have an important function in standardising ethics requirements, but this usually only extends to standardising the consent process. This means that the wider and potentially more important interests of embryo and gamete donors in relation to burdens and possible exploitation are erased from the stem cell banking discourse, and the stem cell banking discourse thus becomes isolated from the more general debate about the flow of reproductive tissues and labour in modern society. Policy developments in the area of reproductive surrogacy seem to indicate an increased focus on the importance of women’s reproductive labour, and the potential for exploitation in relation to reproductive labour (Sugden 2015). Although some in the stem cell field see themselves as outside of the sphere of reproduction and reproductive policy, it is not obvious that society sees it that way. A lack of proper attention to the rights and interests of embryo and gamete donors in relation to stem cell derivation may over time undermine policy support for the field.

## Footnotes

1 In this paper ‘stem cell’ will be used for human embryonic stem cell, unless otherwise specified.

2 “The National Stem Cell Bank will act according to the ethical principles contained in international conventions on human rights and biomedicine to which Spain is a party and in accordance with the principles of objectivity, collaboration, integration and solidarity” (my translation).

3 The creation of embryos also requires the use of male gametes, but there is usually no practical problem involved in procuring sperm and sperm donation does not involve any major burdens for the donor.

4 But, note that the number of spare embryos is not a natural given, but an artefact of IVF practices and human decision-making, and that the very concept of’spare’ is contested, see for instance Holm (1993) and Svendsen and Koch (2008).

5 The potential for exploitation in embryo sharing arrangements will be discussed below in relation to the very similar exploitation issues that arise in relation to the sharing of ova.

6 For an argument that at least one of the standard requirements of the UK Stem Cell Bank is ethically problematic see Holm (2006).

7 The same effect can be achieved if important funding agencies decide only to fund research using cell lines derived according to ethical standards, see for instance National Institutes of Health Guidelines on Human Stem Cell Research (2009).

8 For present purposes it does not matter whether’ necessarily’ is read in an absolute sense, or in a relative sense, as long as the relative necessity is sufficiently strong. For a more detailed analysis of the concept of ‘necessity’ in relation to hESC research see Holm (2003).

9 Facilitating the conduct of high quality stem cell research using existing stem cell lines is, arguably important even if the derivation of stem cell lines is seen as ethically problematic.

10 This can be quite complicated since the original holders of these rights may have sold them on to other parties.

11 http://www.nibsc.org/science_and_research/advanced_therapies/uk_stem_cell_bank/available_human_cell_lines.aspx. Accessed 20.11.14.

12 http://www.nibsc.org/science_and_research/advanced_therapies/uk_stem_cell_bank/policies_and_guidelines.aspx. Accessed 20.11.14.

13 Except for those who hold that foetuses have full or significant moral status and rights.

14 Or strictly speaking 100 (1 – 0.5^x^) % where x is the number of stem cell lines derived. This follows from the fact that each embryo shares 50 % of its genetic material with each of the gamete donors, and from the rules of probability theory.
